# Predicting Antimicrobial Activity at the Target Site: Pharmacokinetic/Pharmacodynamic Indices versus Time–Kill Approaches

**DOI:** 10.3390/antibiotics10121485

**Published:** 2021-12-04

**Authors:** Wisse van Os, Markus Zeitlinger

**Affiliations:** Department of Clinical Pharmacology, Medical University of Vienna, 1090 Vienna, Austria; wisse.vanos@meduniwien.ac.at

**Keywords:** pharmacokinetics, pharmacodynamics, target site, probability of target attainment, time–kill curves

## Abstract

Antibiotic dosing strategies are generally based on systemic drug concentrations. However, drug concentrations at the infection site drive antimicrobial effect, and efficacy predictions and dosing strategies should be based on these concentrations. We set out to review different translational pharmacokinetic-pharmacodynamic (PK/PD) approaches from a target site perspective. The most common approach involves calculating the probability of attaining animal-derived PK/PD index targets, which link PK parameters to antimicrobial susceptibility measures. This approach is time efficient but ignores some aspects of the shape of the PK profile and inter-species differences in drug clearance and distribution, and provides no information on the PD time-course. Time–kill curves, in contrast, depict bacterial response over time. In vitro dynamic time–kill setups allow for the evaluation of bacterial response to clinical PK profiles, but are not representative of the infection site environment. The translational value of in vivo time–kill experiments, conversely, is limited from a PK perspective. Computational PK/PD models, especially when developed using both in vitro and in vivo data and coupled to target site PK models, can bridge translational gaps in both PK and PD. Ultimately, clinical PK and experimental and computational tools should be combined to tailor antibiotic treatment strategies to the site of infection.

## 1. Introduction

The increasing emergence and spread of antimicrobial resistance and the concurrent lack of development of novel antibiotics to counter it represents a global health risk [1,2,3,4,5]. As of September 2020, there were 43 antibiotics in clinical development pipelines, but only two of those belong to novel chemical classes and target multi-drug resistant Gram-negative pathogens categorised as critical by the World Health Organisation [5]. In light of this, it becomes increasingly important that dosing strategies for both existing and newly developed antibiotics are optimised to cure infections efficiently whilst simultaneously minimising the risk of resistance development.

Antimicrobial dosing strategies and clinical susceptibility breakpoints are predominantly set and evaluated based on drug concentrations in plasma. However, the efficacy of antimicrobials is governed by pharmacokinetic/pharmacodynamic (PK/PD) relationships at the infection site, which in most cases is not the bloodstream. Since antibiotics might be distributed unequally between the systemic circulation and different tissues, inferring antimicrobial activity solely from systemic drug concentrations is often not appropriate. Basing dosing strategies on plasma PK data may result in suboptimal or supraoptimal exposure levels at the infection site [6,7], increasing the risk of therapy failure, resistance development, or unnecessary toxicity. This approach can be especially problematic when treating patient populations for which tissue penetration of certain antibiotics has been shown to be impaired or highly variable, such as septic [8,9,10,11,12,13,14,15], other critically ill [16,17], obese [18,19,20,21,22,23,24], diabetic [14,25,26,27], or post-operative patients [28].

The importance of determining PK at the site of infection is now widely acknowledged, and also stressed by regulatory authorities [29,30]. Being the most common foci of infection, drug penetration into the lungs, urinary tract, skin and soft tissues deserve particular consideration [31]. Antimicrobial concentrations in urine, which drive bacterial killing in some types of urinary tract infections [32,33], are easily obtained. Microdialysis [34,35,36,37] and bronchoalveolar lavage [38] are the most common techniques used to sample interstitial space fluid and epithelial lining fluid (ELF), respectively.

However, target site PK should not only be measured, but subsequently needs to be used to estimate potential effects at that target site. This should not be based on the ratio between plasma and target site concentrations at a single time point, neither on the observation that concentrations at the target site exceed the minimum inhibitory concentration (MIC) of relevant pathogens at a certain time point [39], as PK profiles in tissues can have a markedly different shape compared to those in plasma. Rather, information on the entire concentration–time profile at the target site should be used to predict antimicrobial effect.

**Figure 1:**
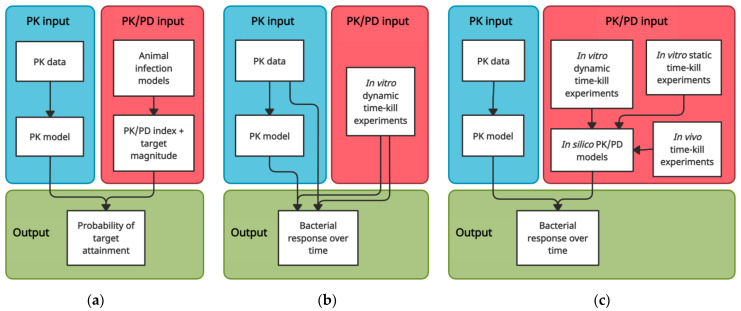
Schematic overview of translational approaches to predict antimicrobial efficacy based on PK data. (**a**) PK/PD index—PTA approach; (**b**) In vitro dynamic time–kill approach; (**c**) Computational PK/PD modelling approaches.

Since following the microbiological course of an infection in a quantitative way in patients is usually not feasible, we rely on the translation of preclinical data to link PK to predictions of antimicrobial effect. One approach that allows for doing so is predicting the probability of target attainment (PTA) based on PK/PD index targets. Alternatively, the time-course of bacterial response to specific PK profiles can be depicted in time–kill curves, which can be established using in vitro, in vivo, and computational modelling approaches (Figure 1). We review these approaches and discuss their strengths, limitations, and opportunities for future research, with a specific focus on their translational capacity from a target site PK/PD perspective.

## 2. PK/PD Index Targets and PTA Analyses

The most frequently used approach to evaluate antibiotic dosing regimens, determine susceptibility breakpoints, or predict whether observed PK profiles are likely to result in sufficient antimicrobial effect involves determining the probability of attaining a PK/PD target. The PK/PD target is usually set based on studies in animal infection models. The different animal infection models used for studying antimicrobial PK/PD and their respective advantages, limitations and challenges have been described by others in detail [40,41,42,43]. Here, we provide a brief general description of the approach (Figure 1a) and mainly focus on the translational capacity of PK/PD targets derived from animal infection models.

### 2.1. Selecting PK/PD Targets and Performing PTA Analyses

The most common infection models to establish PK/PD targets are murine thigh or lung infection models. The mice are often rendered neutropenic to be able to study the drug–pathogen interactions without antimicrobial contributions of the immune system, and are therefore said to be reflective of antimicrobial effect in a worst-case scenario (i.e., in severely immunocompromised patients). After introducing an infection into the tissue, dose fractionation studies are performed, in which a range of total daily doses, given either as a single dose or divided over different numbers of doses, is administered to different groups of animals. Blood samples to determine drug concentrations are taken at regular time intervals. The infected tissue is removed and homogenised at the end of the study period, usually 24 h, and bacterial density (expressed in colony-forming units [CFU]/mL) in these tissues is determined. The antimicrobial effect, defined as the difference in bacterial density in the infected tissue relative to the start of the treatment, is then correlated to three PK/PD indices: the fraction of time the unbound drug concentration exceeds the MIC (*f*T > MIC); the unbound drug concentration area under the curve to MIC ratio (*f*AUC/MIC); and the peak unbound drug concentration to MIC ratio (*f*C_max_/MIC). The PK/PD target is then determined based on the PK/PD index that correlates best with antimicrobial effect and the magnitude of that index required to achieve a certain PD target, e.g., stasis or a 1- or 2-log_10_ reduction in bacterial count in the infected tissue after 24 h of drug exposure [44,45,46].

PK/PD targets have also been derived clinically. However, doing so is complicated due to the fact that usually a single dosing regimen is administered; a dose fractionation design is not possible in the clinic. Moreover, the endpoints in these studies are generally clinical cure and microbiological eradication. These endpoints, despite ultimately being the most relevant for antibiotic therapy, are difficult to measure, not necessarily attributable to the antibiotic effect or the presence of infection, and in the case of clinical cure partially subjective. It may also be difficult to include a sufficient number of patients with the relevant infection types and pathogens, and the range of MICs of these pathogens may be limited. Nonetheless, clinically derived PK/PD targets have generally been in good agreement with those derived from animal models [47]. This has generated confidence in the translational value of PK/PD targets derived from animal infection models.

In a subsequent translational step, PK/PD targets can be combined with observed or predicted human PK profiles to predict whether drug exposure in humans is likely to be effective. The PK input in such analyses usually comes from a population PK model that describes the structural trends and variability in PK data. Using the model, stochastic (Monte Carlo) simulations, which take into account the estimated variability, are performed to generate a large number of PK profiles. Based on these simulations, the probability that a given dosing regimen results in the PK/PD target being attained across a clinically relevant distribution of MICs of the pathogen of interest can be determined [48].

Since PK/PD targets play a central role in the development of antimicrobials and are pivotal in gaining regulatory approval [29,30,49], they are available for the majority of antibiotics. This, together with the fact that PK/PD targets capture the requirements for a PK profile to be deemed effective in a single summary parameter for both PK (e.g., the AUC) and PD (i.e., the MIC), means that PTAs can be calculated in a relatively straightforward, efficient, and economical way once new PK data, such as in special patient populations or certain tissues, become available.

### 2.2. Influence of the Shape of the PK Profile and Study Design

PTAs can be calculated based on summary PK parameters, but it is not necessarily true that antimicrobial effect is solely related to the magnitude of a PK/PD index, regardless of the shape of the PK profile. This was neatly demonstrated by Nielsen et al., who simulated dose fractionation studies using population PK models and PK/PD models based on in vitro time–kill data for six antibiotics of different classes. In their simulations, the selection of the PK/PD index and its target magnitude were sensitive to alterations in PK, e.g., when reduced drug clearance or PK in a different patient population or peripheral compartment were simulated [50]. Dhaese et al. performed a systematic review and meta-regression of data from preclinical infection models, which showed that the mode of infusion of β-lactam antibiotics influences PK/PD target selection [51]. These studies show that a given PK/PD index magnitude may not result in the same antimicrobial effect at all infection sites, due to the different shapes of the PK profile at these sites. Using a single PK/PD target based on plasma PK data may therefore be inappropriate.

The shape of the PK profile potentially affecting PK/PD target selection also has implications for the translatability of PK/PD targets obtained in animal infection models. This is because the shape of the PK profile in animals such as mice and rats may be considerably different from that in humans, as drug clearance in these smaller animals is often much quicker [46,52,53]. Some approaches to correct for this have been described, for example through chemically impairing renal function [54,55,56], or administering small extra drug doses in regular intervals [52,57] or through infusion pumps [58,59,60]. Indeed, Craig et al. showed that in mice with impaired renal function AUC/MIC correlated best with the effect of amikacin against most pathogens, whereas in mice with normal renal function *f*T > MIC was the dominant PK/PD index [54]. However, it should be noted that approaches to prolong drug half lives in animals are not routinely applied in the dose fractionation studies used to determine PK/PD targets.

Study design also impacts PK/PD target selection. Several studies demonstrated that the PK/PD index which correlates best with antibacterial effect depends on which dosing intervals are included in the analysis [46,61,62]. Also, higher inoculum sizes have been shown to require higher PK/PD target magnitudes to achieve the same absolute reduction in CFU counts [63]. This last point may be explained by the higher probabilities of pre-existing or emerging resistant mutants in large bacterial populations, or lower total (due to drug degradation or binding) or per-bacterium (due to larger population size) effective drug concentrations [64].

### 2.3. Plasma PK and Infection Site PD

It is important to realise that in most dose fractionation studies, plasma PK is linked to bacterial kill at the site of infection. This may limit the translational capacity of PK/PD targets in two additional ways.

First, drugs distribute unevenly between plasma and different tissues. The selected PK/PD target is thus dependent on the type of infection model used. The limitations associated with this are well illustrated in a study by Maglio et al., which found bactericidal effects of clarithromycin in the mouse lung model but not in the mouse thigh model, most likely resulting from intrapulmonary accumulation of the drug [65]. Therefore, basing clarithromycin PK/PD targets and susceptibility breakpoints on the mouse thigh model would result in overly pessimistic susceptibility breakpoints and an overestimation of the PK/PD target required in plasma to eradicate infections in the lung. The authors note that this might explain the lack of observed clinical failures with macrolides in the treatment of pneumonia, despite pathogens being classified as resistant [65].

Second, tissue distribution in humans may deviate from that in animal infection models. Therefore, even if a relevant infection model is used, the link between plasma PK and antimicrobial effect at the infection site, and thus the PK/PD target found in these studies, may not be directly translatable to humans. For example, a study by Rodvold et al. found that ceftobiprole penetration from plasma into ELF was much higher in mice than in humans. In other words, a given drug exposure in plasma results in much lower ELF exposure in humans than in mice for this drug, and using the PK/PD target based on mouse plasma PK to guide dosing strategies for treating pneumonia would likely result in insufficient lung exposure in patients [66].

For these reasons, it makes sense to characterise not only bacterial response but also PK at the infection site, so PK/PD targets are based on a direct link between PK and PD [38,67,68,69]. Several studies have linked drug exposure in ELF to bacterial kill in a murine lung model to establish PK/PD targets specific for the treatment of pneumonia [66,70,71]. Ideally more such site-specific PK/PD targets would be established, especially for antibiotics that distribute unequally across different tissues or for infections in sites to which distribution may be hampered or at which accumulation might occur, such as meningitis, pneumonia, osteomyelitis, or infections caused by intracellular pathogens [30,42,72,73]. Doing so adds to the complexity of the experimental procedures, but it increases the translational value of these targets, and the confidence we can have in outcomes of PTA analyses using human target site PK. However, for some infection types, such as lower urinary tract infections or complicated intra-abdominal infections, the animal infection models with which PK/PD targets can be established do not yet exist [41].

### 2.4. Informative Value of PD Endpoints

PK/PD targets are selected based on total bacterial counts measured at a single time point, generally after 24 h of drug exposure. A major limitation of this approach is that a single measurement provides no information on the time-course of effect, the rate and extent of kill, and the trend in bacterial population size at the time of measurement.

Moreover, potential resistance development is often ignored in dose fractionation studies, and the short study duration in combination with the single measurement of bacterial density means potential regrowth due to amplification of resistant subpopulations can often not be captured. However, drug exposure relates differently to the emergence of resistant subpopulations than to bacterial kill [63,74,75,76,77]. Drug exposures resulting in net bacterial kill during the first 24 h, and thus appearing effective, may actually provide the selection pressure that drives the development of resistance [74]. Higher PK/PD index magnitudes have been found to be required for resistance suppression than for reductions in bacterial count, with correspondingly lower target attainment rates for resistance suppression targets in PTA analyses [63,70]. It should be noted that the shape of the PK profile may also affect the development of resistance. In particular, slowly increasing antibiotic concentrations, as sometimes observed in peripheral tissues, may select for less susceptible subpopulations [78], promote the development of adaptive resistance [78,79] or the formation of persister cells [80].

### 2.5. Reliance on the MIC

All PK/PD indices include the MIC as a means to link PK to antimicrobial effect. However, the MIC is a measure with major limitations, including the static drug concentration under which it is measured, the poor precision and upward bias stemming from the twofold dilution steps used in its determination, and considerable assay variation [81,82]. Moreover, the endpoint on which the MIC is based, the presence of visible growth after overnight incubation, is also based on a single measurement and is additionally highly uninformative due to its dichotomous nature and the fact that growth media starts to become visibly turbid only at bacterial concentrations of 10^7^–10^8^ CFU/mL [82]. By relying heavily on the MIC, these limitations are propagated into the PK/PD indices and the PTA analyses based on PK/PD index targets.

## 3. Time–Kill Curve Approaches

Time–kill curve approaches represent alternatives to the PK/PD index approach to investigate antimicrobial PK/PD. In time–kill experiments, bacteria are inoculated in an experimental system and exposed to an antibiotic. Through periodic sampling time–kill curves can be established, depicting bacterial concentrations over time. In this section, we discuss how in vitro, in vivo, and in silico time–kill curve approaches can be used as a translational tool to investigate antimicrobial PK/PD, again with a focus on target site PK/PD.

### 3.1. Time-Course of Effect

A common advantage of time–kill approaches is that they allow for the evaluation of the complete PK/PD time-course, which overcomes many limitations related to the sequential workflow and dependence on summary endpoints of the PK/PD index approach [83,84,85,86,87,88]. Different antibiotics, pathogens, dosing regimens, or shapes of PK profiles can be compared in much more detail, even if these profiles have similar PK/PD index magnitudes and thus would be deemed equally effective in conventional PTA analyses. By plating samples on drug-containing agar plates, the emergence of less susceptible subpopulations can also be followed over time.

### 3.2. In Vitro Time–Kill Experiments

Most time–kill experiments are performed in vitro. A classification and detailed overview of the many different types of time–kill setups and their respective advantages and disadvantages has been published by Gloede et al. [89]. Broadly, they can be divided into static systems, in which drug concentrations are often assumed to be constant and no media exchange occurs, and dynamic systems, in which drug concentrations are made to change over time and fresh media is exchanged with or supplied to the culture.

#### 3.2.1. Simulating Target Site PK

Data from static time–kill systems are useful in computational PK/PD modelling and can be used to answer specific pharmacodynamic questions, e.g., on the impact of specific pathophysiological conditions on PK/PD. However, they are not representative of the clinical situation from a PK perspective. In dynamic time–kill systems, on the other hand, any clinical PK profile can be precisely mimicked. This makes dynamic time–kill experiments suitable for simulating bacterial response to PK observed in specific tissues (Figure 1b). Indeed, setups of varying complexity have been used to simulate target site PK/PD of antimicrobials, ranging from simple stepwise dilution approaches [8,11,90,91,92,93,94,95,96,97], to more advanced continuous dilution methods [98,99,100,101,102,103,104,105,106,107,108,109], and setups in which drug and media diffuse through membranes to a peripheral compartment containing the bacteria [110,111,112,113,114,115,116,117], such as the hollow fibre infection model [118]. Various setups that aim to mimic specific target site PK characteristics have also been described [87,89]. For example, in vitro bladder infection models, pioneered by O’Grady et al., can be used to simulate the continuous filling and periodic emptying of the bladder [119,120,121,122].

Dynamic time–kill setups allow for flexibility in experimental design, not only in terms of the PK profile to simulate but also in terms of the initial inoculum size, for example. However, the number of experiments that can be feasibly performed is limited. Setups such as the hollow fibre infection model are particularly resource intensive and time consuming. So, especially when no subsequent computational PK/PD modelling is planned, the experimental conditions should be carefully selected. By far the most common approach in dynamic time–kill experiments is to simulate typical PK profiles. However, by doing so, the variability in PK between patients is ignored. This is particularly relevant when tissue PK is simulated, as tissue exposure often varies widely between patients. The lower end of the drug exposure distribution, in particular, deserves consideration in dynamic time–kill experiments, since we are generally more interested in assessing whether patients with low drug exposures face risk of treatment failure, and in vitro we cannot evaluate toxicity that might occur in patients with high tissue drug concentrations. For example, Hamada et al. simulated in a hollow-fibre infection model the 10th, 25th and 50th percentile of soft tissue exposure levels attained after a common dosage of vancomycin [106], an example of a drug for which in several different patient populations tissue exposure has been shown to be highly variable [13,25,123,124,125,126].

#### 3.2.2. Translational Capacity of In Vitro Environment

The vast majority of in vitro PK/PD studies are performed in growth media such as Mueller–Hinton broth. Using standardised growth media enhances reproducibility, facilitates comparison between studies, and allows for assessing the interaction between drug and pathogen without potentially confounding factors. However, it has long been appreciated that the infection site environment plays a role in bacterial growth and antimicrobial activity [99]. Host factors such as physiological proteins and immune system components are absent in these growth media. Additionally, the pH value, which widely varies between different body fluids, may affect antimicrobial effect, and drug exposure may be different in homogeneous suspensions compared to the complex infection site matrix with anatomical barriers in vivo [89]. Moreover, bacteria grown in nutrient-rich growth media may be phenotypically different compared to bacteria grown in vivo [127], and growth rates are generally much higher in vitro [127,128,129]. Static time–kill systems have additional limitations. The lack of fresh media supply may result in nutrient depletion and (toxic) waste product accumulation, and the bacteria are exposed to non-dynamic and sometimes clinically irrelevant drug concentrations. These factors may impact bacterial response both phenotypically and genotypically [130]. Moreover, the duration of static time–kill experiments is usually restricted to 24 h, which may be too short to capture regrowth due to resistance development.

Thus, despite the fact that in vitro dynamic time–kill studies can be used to simulate human PK, results should not be directly translated to humans. However, these studies are useful when comparing different drugs and dosing regimens, and evaluating the effect of antibiotic combination treatment at clinically relevant PK.

Performing time–kill studies in in vitro environments that mimic the infection site theoretically makes sense to improve their translational value. However, a recent review indicated that research on antimicrobial PD in body fluids (e.g., urine, bile fluid, or cerebrospinal fluid) or growth media supplemented with human material (e.g., serum or albumin) remains scarce [131]. This is most likely due to limitations related to variability and availability. The impact that substituting standardised growth media for adapted media or body fluids has on antimicrobial activity varies between strains, antimicrobial agents, and the fluids used [131]. Moreover, the composition of a given body fluid can vary significantly between individuals and within individuals at different time points, e.g., based on diet, which may affect bacterial growth and antimicrobial effect. For example, we recently found large variability in in vitro bacterial growth and ceftriaxone effect in individual ascitic fluids from ten different ascites patients [132]. There are practical limitations too, as obtaining body fluids may involve challenging or invasive procedures, and extracting it in sufficient quantities might not be feasible. This last point renders performing experiments with dynamic setups that require a considerable amount of media challenging or impossible with certain pure body fluids.

### 3.3. In Vivo Time–Kill Experiments

Time–kill experiments in animal infection models are a seemingly attractive alternative to their in vitro counterparts, as studying PK/PD in an in vivo infection site environment has obvious advantages. When it comes to translational value from a PK perspective, however, in vivo time–kill experiments suffer from similar limitations as the dose fractionation studies used to determine PK/PD targets.

Time–kill experiments in rodents follow the same workflow as the dose fractionation studies described in Section 2.1, with the difference that bacterial counts are measured at various time points, rather than only at the end of the experiment [54,129,133,134,135]. However, each individual animal can only contribute data for a single time point, as quantifying bacterial counts requires the infected tissue to be removed and the animal in question to be sacrificed. Moreover, since this means that the course of the infection cannot be followed within the same animal, the sacrifice of multiple animals per time point is necessary to account for the variability in PK and PD between animals. The frequent sampling that renders time–kill curves valuable therefore requires the sacrifice of large numbers of animals, which raises ethical concerns and explains in part why time–kill experiments in rodents are not often performed.

### 3.4. PK/PD Modelling of Time–Kill Data

Data from time–kill experiments can be used to inform PK/PD models, which provide a quantitative description of bacterial growth and drug effect. These models are usually semi-mechanistic or mechanism-based, which means that their structure can be based on knowledge of, for example, the mechanism of action of the antibiotic, or the various mechanisms by which a pathogen may acquire reduced susceptibility to it [83,86].

#### 3.4.1. Simulating Target Site PK/PD

With drug effect and bacterial growth described in equations, a PK/PD model can be used to simulate bacterial response to untested scenarios. For example, PK/PD models based on in vitro static time–kill data have shown the ability to predict bacterial response under dynamic drug concentrations [136,137] and across strains with different drug susceptibilities, as well as the inoculum effect [138].

The flexibility and extrapolation capacities of computational PK/PD models render them useful for detailed comparisons of drugs and dosing regimens or informing the design of further preclinical experiments or clinical trials. It also makes them particularly useful to predict PK/PD at different infection sites, since there can be large differences between different tissues in terms of PK but also in terms of the typical bacterial burden of an infection [139]. Several studies have linked models describing PK in specific body compartments to PK/PD models in order to simulate bacterial response over time at the target site (Figure 1c) [97,140,141,142,143,144,145]. In this way, comparisons in terms of target site antimicrobial effect have been made between antibiotics [145], dosing regimens [140,141,143,145], drug formulations [141], and infection sites [142,143,144].

However, few target site-specific time–kill simulations have been performed with PK models that include a measure of variability around PK parameters [143,144]. As discussed, the between-patient variability in peripheral tissue PK is often significant and it is thus important that this is reflected in stochastic time–kill curve simulations. Besides variability in PK, the variability in bacterial response as observed in the time–kill experiments (e.g., within strains or between strains with the same MIC) can also be taken into account in the simulations [143,144]. This is an advantage over conventional PTA analyses using a PK/PD index target, in which only the variability in PK is considered.

PK/PD model simulations can be used to calculate time–kill curve-based PTAs and define breakpoints. For example, Iqbal et al. linked a PK model describing plasma, muscle, and subcutaneous PK in sepsis patients to a PK/PD model developed using in vitro static time–kill data to predict target site-specific breakpoints for stasis, 2-log_10_ kill, and resistance development suppression for moxifloxacin against *E. coli* and *Staphylococcus aureus* [143]. To allow the prediction of time–kill curves for strains with different susceptibilities, the model included the MIC as a covariate on drug effect parameters. Due to the relatively similar moxifloxacin PK observed in plasma, skin, and subcutis, the breakpoints for the different infection sites were generally the same. However, as the authors note, this may not be the case for drugs that display impaired or delayed distribution to the target site, or where target site PK is associated with high variability [143]. As this study illustrates, to inform clinical dosing strategies time–kill curve approaches also rely on easily obtainable susceptibility measures such as the MIC. In contrast to the PK/PD index approach, however, time–kill curve-based PTA analyses and breakpoint selection take into account the entire time-course of and variability in PK and PD simultaneously.

The PK input in simulations of bacterial response to human drug exposure often comes from population PK models developed using a mainly data-driven (‘top down’) approach; the optimal model structure and parameter estimates are based on the fit of the observed PK data. When it comes to predicting the efficacy of antibiotics in different tissues or when no clinical PK data is available, physiologically-based (PB) PK models offer a notable alternative. These models employ a more mechanistic (‘bottom up’) approach to predicting drug disposition to different tissues by using a compartmental structure that is based on anatomical and physiological characteristics of the body [146,147]. The utility of such models in predicting target site PK/PD was shown by Sadiq et al., who developed a whole-body PBPK model for ciprofloxacin based on plasma concentrations and plasma-to-tissue distribution coefficients from the literature to predict typical PK profiles in a variety of tissues [142]. This PBPK model was subsequently linked to a PK/PD model by Khan et al. [148] to predict the effect of ciprofloxacin against several *E.coli* isolates in the different tissues. Predicted drug exposures, and, accordingly, bacterial killing, varied widely between tissues. Such a modelling framework can be used to predict the time-courses of antimicrobial effect in different tissues and identify sites where an antibiotic may display strong activity, or lack thereof [142].

PK/PD modelling and simulation approaches also have great value in the translation of the effect of antibiotic combination therapies. Drug interaction effects depend on the concentrations of the individual antibiotics and the ratio between them, and consequently on the dosing amounts and intervals of each drug and the specific shape of the PK profiles [149]. Due to the many dosing designs that need to be considered and inter-species differences in PK, dose fractionation studies in animals are not well suited to investigate optimal dosing strategies of antibiotic combinations. Moreover, the summarised nature of PK/PD index targets fails to account for the PK profile-specific dynamic changes in the concentrations of the drugs and the ratio between them [83]. The hollow fibre infection model is often used to investigate drug combinations [150,151,152,153,154], but testing all potentially clinically relevant dosing strategies is impractical. In PK/PD models, in contrast, drug interactions can be described in equations based on limited experimental data and, through simulations, optimal dosing strategies can be identified [133,149,155,156,157,158]. Again, these simulations should be based on PK at the target site as this is where the drug interaction is relevant.

#### 3.4.2. Translational PK/PD Modelling

Most PK/PD models of antimicrobials are based on in vitro static time–kill experiments in rich growth media. These experiments provide a cheap, practical, and relatively easy way to assess bacterial response to a wide range of drug concentrations, which is useful for estimating drug effect parameters in PK/PD models. However, they are poorly reflective of the infection site environment in vivo. It should be noted that despite this limitation, PK/PD models based on static time–kill experiments have shown the capacity to predict time–kill under dynamic drug concentrations [136,137], as well as PK/PD index targets derived from animal infection models [50,62,136,158,159]. The fact that PK/PD models can be based on simple static time–kill experiments requiring small volumes of growth media also presents the opportunity to develop translational PK/PD models based on experiments in human body fluids or adapted growth media.

Two important strengths of translational modelling and simulation are its ability to inform the optimal design of further preclinical or clinical studies, and its potential to integrate data from multiple sources. For example, simulations using PK/PD models developed based on static time–kill data can be used to select PK profiles to test in dynamic time–kill experiments, data from which can subsequently be used to validate the model structure or improve parameter estimation [83,137]. Efforts have also been made to include the effect of the immune system on bacterial kill in PK/PD models [142,160], based on data from murine infection models, for example [161,162,163]. To fully leverage these strengths, however, it is important that data are compatible (e.g., by using the same bacterial strains), and close coordination between research disciplines is therefore required.

Integrating in vivo time–kill or efficacy data in the model development process not only increases the translational power of model predictions, but also maximises the value of sparse animal data as inter-species differences in PK are accounted for, which is desirable both from a scientific and ethical perspective. A recent work by Sou et al. provides an example of how sparse in vivo efficacy data in combination with PK/PD modelling can provide a translational step between in vitro PK/PD and predicting clinical efficacy. The authors developed a semi-mechanistic PK/PD model for apramycin, a novel aminoglycoside antibiotic, against *E. coli* based on in vitro static time–kill data of four strains. Data obtained in a murine thigh infection model with the same strains were subsequently used to correct for the slower growth rate observed in vivo, and final model parameters were re-estimated using both in vitro and in vivo data. This final PK/PD model was then coupled to a human apramycin PK model to calculate a human efficacious dose [61]. As with the PK/PD index methodology, for certain infection types or antibiotics it is important to measure drug concentrations at the infection site to optimise the translational value of in vivo data in PK/PD models. For example, Lin et al. developed an in vivo semi-mechanistic PK/PD model, the structure of which was based on a model developed with in vitro static time–kill data, for aerosolised colistin against *Pseudomonas aeruginosa* using time–kill data from a murine lung infection model and a population PK model describing ELF and plasma PK in mice [134]. The PK/PD model was subsequently linked to a PK model describing colistin concentrations in ELF of critically ill patients [164] in order to predict an optimal clinical dosing regimen [134].

## 4. Conclusions

We have discussed the capacity of different translational approaches to predicting antimicrobial efficacy at the target site. It is clear that each approach has both strengths and drawbacks (Table 1). PK/PD index targets are based on antibacterial effect in vivo, and PTA analyses using these targets allow for estimating efficacy across MICs in an economic way. However, PK/PD targets are often based on plasma PK in rodents, and differences in drug distribution and PK profile shape between species and tissues are ignored. Moreover, they are fixed summary endpoints that provide no detailed information on antimicrobial effect and often do not consider resistance development. Time–kill experiments, in contrast, allow the evaluation of the complete PK/PD time-course, including resistance development. On their own, however, these approaches lack translational power due to the experimental environment (in case of in vitro dynamic time–kill experiments) or inter-species differences in PK (in case of in vivo time–kill experiments). Additionally, a limited number of conditions can be investigated. PK/PD models can optimally bridge translational gaps in both PK and PD through integrating different sources of data and allow for simulating untested scenarios. We believe that combining PK/PD models based on both in vitro and in vivo (time–kill) data with target site PK models is the most powerful approach to optimise antibiotic dosing regimens.

Ultimately, however, the choice of translational approach should be based on the primary objective of the analysis and the available information on PK and PD. For example, if rapid equilibration between plasma and target site concentrations is observed for a time-dependent antibiotic, current dosing strategies are likely appropriate to treat infections at the site in question (provided that plasma concentrations of this drug are known to be efficacious). In such cases, PTA analyses are valuable if large variability in target site concentrations between individuals is observed, but laborious in vitro or in silico simulations of target site PK/PD may have limited added value. The value of time–kill approaches increases, however, if the shape of the target site PK profile is very different to that in plasma or other tissues, or if impaired distribution to or accumulation at the target site is observed. When it comes to novel antibiotics, model-based translation should play a central role in de-risking and accelerating the development process. PK/PD models quantify concentration–effect relationships early on and can thereby inform the design of further translational research. These models can continuously be improved as data from increasingly complex systems become available, and ultimately guide the design of optimal dosing strategies [83]. Moreover, in the absence of clinical PK data, model-based approaches can be used to predict target site concentrations, e.g., through PBPK modelling. Such predictions may serve as the basis for studies in hollow-fibre infection models. If the antibiotic in development belongs to a well-known class of antibiotics, reasonable predictions of PK/PD relationships and tissue distribution in animals and humans can be made. In such cases, available models of related compounds can play a role in reducing the amount of preclinical (target site) PK and PK/PD work needed [61] and streamline the development of the new compound.

In conclusion, in order to make the best use of available and newly developed antibiotics, improve patient outcomes, and limit resistance development, we should move away from plasma PK-based dosing strategies. Instead, clinical target site PK data and the experimental and computational tools available to us should be leveraged to tailor antibiotic treatment strategies to the site of infection. 

## Figures and Tables

**Table 1:** Strengths and limitations of translational approaches for antibiotic PK/PD.

	PTA Analyses Using PK/PD Index Targets		In Vitro Dynamic Time–Kill Experiments		In Vivo Time–Kill Experiments		Computational PK/PD Modelling	
	+	−	+	−	+	−	+	−
**PK aspects**	Variability in PK is considered	PK/PD target set based on animal PK Based on plasma PK Assumes equal tissue distribution in animals and humans Ignores shape of PK profile	PK profiles can be exactly mimicked	Variability in PK is often not considered	-	Animal PK drives effect Often plasma PK is linked to drug effect	Bacterial response to any PK profile can be simulated Variability in PK can be taken into account	-
**PD aspects**	PK/PD target set based on in vivo data Can be calculated across MICs	PK/PD target set based on PD readout at 24 h Provides no detailed PD information (e.g., rate or extent of kill) Resistance development usually not considered Relies heavily on MIC Within-MIC variability in PD is not considered	Depicts PD time-course Resistance development can be followed	Performed in in vitro environment	Performed in in vivo environment Depicts PD time-course Resistance development can be followed	PD time-course cannot be followed within the same animal	Depicts PD time-course Resistance development can be simulated Variability in PD can be considered Data from multiple sources (e.g., in vivo) can be incorporated	Mostly based on in vitro data, often from static time–kill experiments Dependent on MIC to predict effect on untested strains
**Practical aspects**	PK/PD targets are available for most antibiotics Economical approach	If no PK/PD target is available, animal studies must usually be performed	Can be performed without a PK model	Setups can be complex and resource-intensive Limited number of strains, PK profiles and conditions can be investigated	Can be performed without a PK model	Requires large number of animals, ethical considerations Resource-intensive Limited number of strains, doses and conditions can be investigated	Can be based on data from simple and cheap experiments Untested scenarios can be simulated; these can also inform optimal design of further studies	May be complex and time-consuming Dependent on availability and quality of experimental data
